# Reflections on a seminal article on malnutrition published in the *British Journal of Nutrition*, 2004

**DOI:** 10.1017/S0007114522001155

**Published:** 2022-06-14

**Authors:** Marinos Elia, Rebecca J. Stratton

**Affiliations:** Faculty of Medicine, University of Southampton, Southampton General Hospital, Southampton SO16 6YD, UK

**Keywords:** Malnutrition, Screening, Validity, Adults

Fig. 1 shows the abstract and the authors of a clinical article published in the November issue of the British Journal of Nutrition (BJN), 2004^(1)^. It addressed aspects of malnutrition in inpatients and outpatients at Southampton General Hospital, using the ‘Malnutrition Universal Screening Tool (‘MUST’), which was launched the previous year^(2)^. To understand why the article became highly cited, it is necessary to briefly consider certain events leading up to and following the publication of the article.

**Fig. 1.**
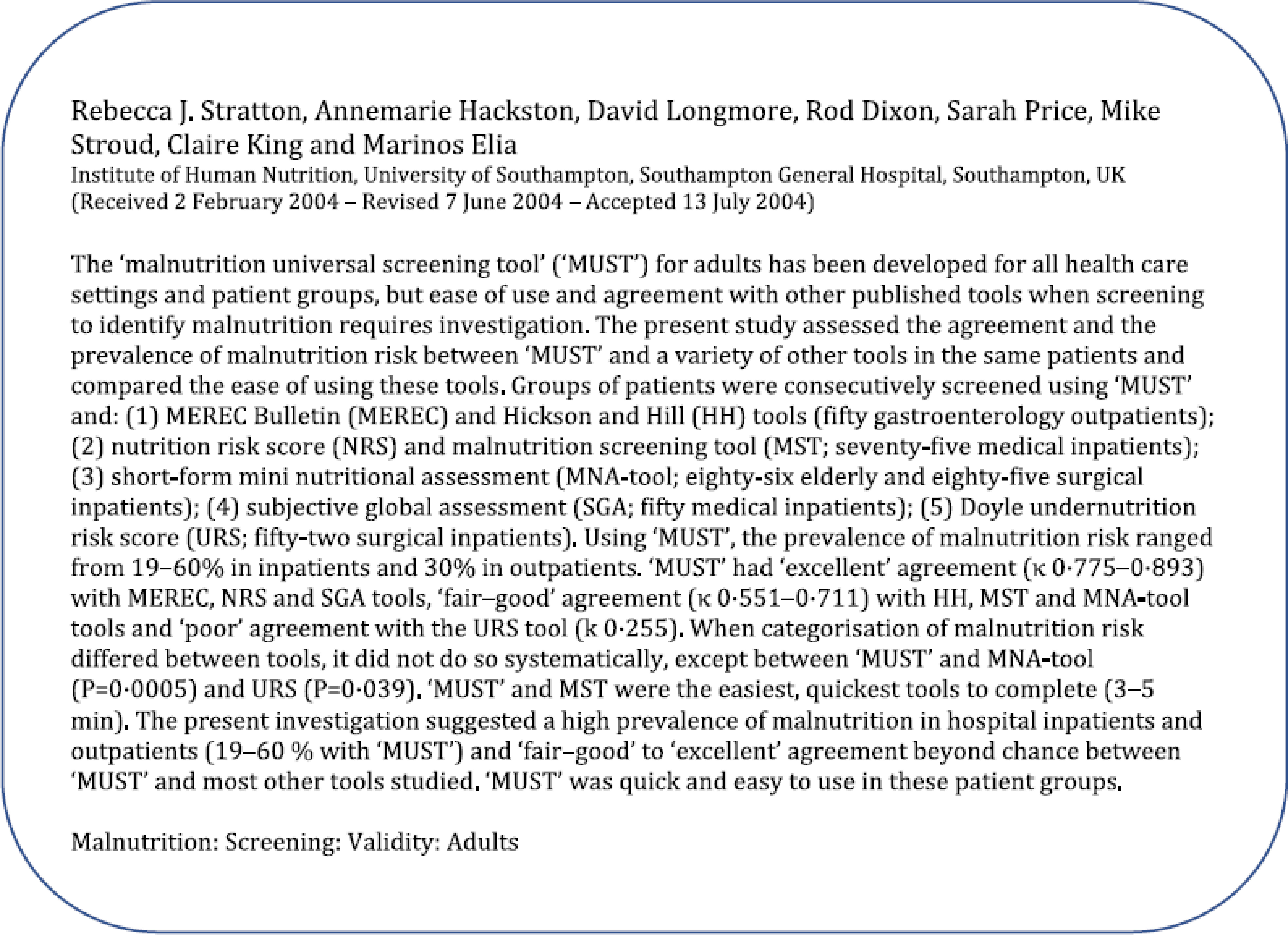
Authors and abstract of the 2004 BJN article.

The British Association for Parenteral and Enteral Nutrition was formed in 1992, with the primary aim of improving the nutritional care of people at risk of malnutrition in various settings. This multidisciplinary organisation, which included doctors, nurses, dietitians, pharmacists and patients, enthusiastically set out to examine the burden of malnutrition in the UK. However, it struggled to do this, not least because there was no universally accepted definition of malnutrition (a continuing problem^(3)^) and nutrition screening tools were based on different criteria, developed for different purposes, age groups and settings^(4,5)^. Screening was either not undertaken or undertaken haphazardly, often with tools that were unreliable, unvalidated and sometimes lengthy and not user-friendly. Furthermore, since some screening instruments were developed for use in only one care setting, or for specific condition(s) and age group(s) (e.g. ≥65 years), they could not be used universally for clinical or surveillance purposes, either within the same care setting or during the patient journey between care settings. And since the criteria used by different tools varied, concordance between them could be poor. A patient’s nutritional status could ‘change’ during their transfer from one hospital ward to an adjacent ward using a different screening tool. There was clearly a need to improve nutritional screening and care.

After working on such problems for several years, the Malnutrition Advisory (latterly, Action) Group of British Association for Parenteral and Enteral Nutrition, launched the ‘MUST’ tool together with its influential report, ‘The “MUST” report’, which defined malnutrition and produced an extensive database of the physiological and clinical principles underpinning the new tool^(2)^. ‘MUST’ combines categories of percent unintentional weight loss in the preceding 3–6 months (the past), current weight status according to the BMI (the present) and the likely forthcoming direction of change (the future – e.g. patients who are persistently unconscious or have persistent swallowing difficulties are likely to become malnourished without treatment). It allows nutritional screening to be undertaken in all care settings on all types of adult patients, even in those who are unconscious, unable to be weighed or have their height measured. It uses objective criteria whenever possible and subjective criteria when necessary, and it links screening results to management plans. However, there was little information in peer reviewed journals about the use of ‘MUST’ in any setting, until the publication of the BJN article.

## The BJN article

The article^(1)^ highlighted three issues associated with the management of malnutrition in hospital. First, it established nutritional status of 348 hospital consecutively admitted inpatients in medical, surgical, specialist surgical (gastrointestinal) and elderly care wards, as well as 50 consecutive patients attending a gastroenterology outpatient clinic. It found 19–60 % of the inpatients and 30 % of the outpatients were at risk of malnutrition (medium and high risk). It reinforced the view that malnutrition was a common and widely distributed clinical problem in hospital. Since nutritional screening was often not undertaken, much malnutrition went undetected and untreated, at great cost to the patients and care services.

Second, in the absence of a gold standard for malnutrition screening, the study established the concurrent (correlational) validity between ‘MUST’ and other screening tools. In twelve paired comparisons between ‘MUST’ and seven other tools in various wards and an outpatient clinic, there was agreement in 67–92 % of patients and chance adjusted agreement in 25·5–89·3 % (*κ*, 0·255–0·893; i.e. from ‘poor’ to ‘excellent’ agreement). Furthermore, with ‘MUST’, the prevalence of malnutrition was significantly lower than with the undernutrition risk score (URS) tool in general surgical wards (19 % *v*. 35 %). Compared with the mini nutritional assessment tool (MNA, short form), the prevalence according to ‘MUST’ was significantly higher in gastrointestinal wards (47 % *v*. 60 %) but significantly lower in elderly care wards (44 % *v*. 85 %). From this study alone, it is difficult to establish the superiority of one tool over another, and therefore screening tool selection needs to consider other issues^(5)^. Among these are: tool validity and reliability, which were absent or inadequate for some tools; the age group(s), setting(s) and condition(s) for which tools were developed; the use of predominantly objective or subjective criteria and the ease and speed of screening. Certain tools incorporated puzzling criteria. For example, in the URS tool increased appetite and constipation were risk factors for malnutrition. However, in patients with disease-related malnutrition, there is usually loss of appetite rather than increased appetite, and in those with inflammatory bowel disease, there is typically diarrhoea rather than constipation.

Third, the study found ‘MUST’ more user-friendly than other tools. It was ‘easy’ or very easy’ to use, taking 3–5 min to complete, whereas other tools were more difficult, taking 5–10 min to complete. These are important considerations in screening tool selection for busy hospital wards and outpatient clinics. Linking the results of screening to a care plan, an integral part of ‘MUST’ but not of other tools, is another important practical consideration.

### What happened next?

The article became highly cited for a variety of reasons, many of which were related to the quest to develop and routinely use a practical national organisational infrastructure to improve the detection, treatment and monitoring (or surveillance) of malnutrition, using sound and consistent criteria for clinical and public health purposes. It was realised that it would be difficult to implement policies, examine trends over time and make interinstitutional comparisons without using consistent criteria that applied within and between care settings and during the patient journey from one care setting to another. The BJN article was one of the first articles to illustrate that malnutrition could be detected and managed using the ‘MUST’ framework in both hospitalised patients and free-living community patients attending outpatient clinics. Apart from emphasising that malnutrition was a common clinical problem, it offered a management plan (low risk, routine care; medium risk, observe; high-risk treat; according to the ‘MUST’ guidance notes). Within a few years ‘MUST’ became the most commonly used screening tool in hospital, community and care home settings, eclipsing all previous screening tools in the UK (also Republic of Ireland)^(5,6)^. Promotion of the ‘MUST’ framework and associated publications, such as the BJN article, helped increase awareness and interest in the burden of malnutrition and its management. At the time of its launch, ‘MUST’ was already endorsed by multiple organisations (British Association for Parenteral and Enteral Nutrition, British Dietetic Association, Royal College of Nursing and Registered Care Home Association), but support grew to include additional Royal Colleges (e.g. Royal College of Physicians, Royal College of General Practitioners) and other governmental and non-governmental organisations. Many national policies and reports on malnutrition, based on the ‘MUST’ framework, were launched, some in the House of Commons with ministerial support. The National Institute for Health and Care Excellence produced guidelines on nutritional support in adults^(7)^ (which cited the BJN article) and a quality standard on nutritional support^(8)^, both of which accepted and used the ‘MUST’ framework. Economic reports on malnutrition and its treatment, published by National Institute for Health and Care Excellence and other organisations, were also based on the ‘MUST’ framework. Furthermore, government reports and commercial companies producing products for nutritional support supported the use of the ‘MUST’ framework, while drawing attention to publications on the prevalence and management of malnutrition. To combat high workloads in clinical practice, a prototype automated electronic system for MUST screening was recently used in outpatients. It reduced screening time from 3 to 5 min, at the time publication of the BJN article, to well under a minute^(9)^.

## Conclusion

The BJN article on malnutrition in hospitals both contributed and resulted from systematic efforts to improve detection and treatment of malnutrition in the UK. Although nutritional care has improved since the publication, malnutrition remains an important clinical and public health problem. Much work still needs to be done including the following: increase awareness about the burden of malnutrition, which continues to be under-recognised and under-treated; support and facilitate continuity of care during the patient journey from one setting to another and encourage self-screening, especially since face-to-face consultations have declined during the ongoing COVID pandemic.

## References

[1] Stratton RJ, Hackston A, Longmore D, et al. (2004) Malnutrition in hospital outpatients and inpatients: prevalence, concurrent validity and ease of use of the 'malnutrition universal screening tool’ ('MUST') for adults. Br J Nutr 92, 799–808. 15533269 10.1079/bjn20041258

[2] Elia M (2003) The 'MUST’ Report. Nutritional Screening for Adults: A Multidisciplinary Responsibility. Development and Use of the 'Malnutrition Universal Screening Tool’ ('MUST') for Adults. A Report by the Malnutrition Advisory Group of the British Association for Parenteral and Enteral Nutrition. Redditch: BAPEN.

[3] Elia M (2017) Defining, recognizing, and reporting malnutrition. Int J Low Extrem Wounds 16, 230–237. 29145755 10.1177/1534734617733902

[4] Elia M & Stratton RJ (2012) An analytic appraisal of nutrition screening tools supported by orginal data with particular reference to age. Nutrition 28, 477–494. 22386636 10.1016/j.nut.2011.11.009

[5] Elia M & Stratton RJ (2011) Considerations for screening tool selection and role of predictive and concurrent validity. Curr Opin Clin Nutr Metab Care 14, 425–433. 21832898 10.1097/MCO.0b013e328348ef51

[6] Russell CA & Elia M (2011) Nutrition Screening Survey in the UK and Republic of Ireland in 2010. A Report by BAPEN. BAPEN. Print edition published February 2011. ISBN 978 1 899467 66 2. https://www.bapen.org.uk/.

[7] NICE (2006) CG32 Nutrition Support in Adults. http://guidance.nice.org.uk/CG32/NICEGuidance/pdf/English (accessed April 2022).

[8] NICE (2012) QS24 Quality Standard for Nutrition Support in Adults. http://publicationsniceorguk/quality-standard-for-nutrition-support-in-adults-qs24 (accessed April 2022).

[9] Elia M, Cawood AL, Akbar T, et al. (2019) Nutritional self-screening in <1 min: evaluation of a measuring station using sonic measurement of height. Nutrition 67–68, 110529. 10.1016/j.nut.2019.06.010 31473522

